# Halo angiokeratoma

**DOI:** 10.1016/j.jdcr.2024.06.010

**Published:** 2024-06-21

**Authors:** Mohammed Ibrahim AlJasser

**Affiliations:** College of Medicine, King Saud bin Abdulaziz University for Health Sciences, Riyadh, Saudi Arabia; King Abdullah International Medical Research Center, Riyadh, Saudi Arabia; and Division of Dermatology, Ministry of National Guard Health Affairs, Riyadh, Saudi Arabia

**Keywords:** angiokeratoma, cherry angioma, depigmentation, dermatoscopy, halo nevus, halo phenomenon, Sutton nevus, vascular lesion, vitiligo

## Introduction

Halo nevus (HN) (also known as Sutton nevus) is characterized by circumferential depigmentation around a melanocytic nevus. It is observed in about 1% of individuals with an onset usually early in life.^1^ HN is associated with different types of melanocytic nevi. The development of the halo phenomenon around nonmelanocytic lesions is rarely reported. We describe a rare occurrence of depigmented halo around an angiokeratoma (halo angiokeratoma).

## Case report

**Fig 1:**
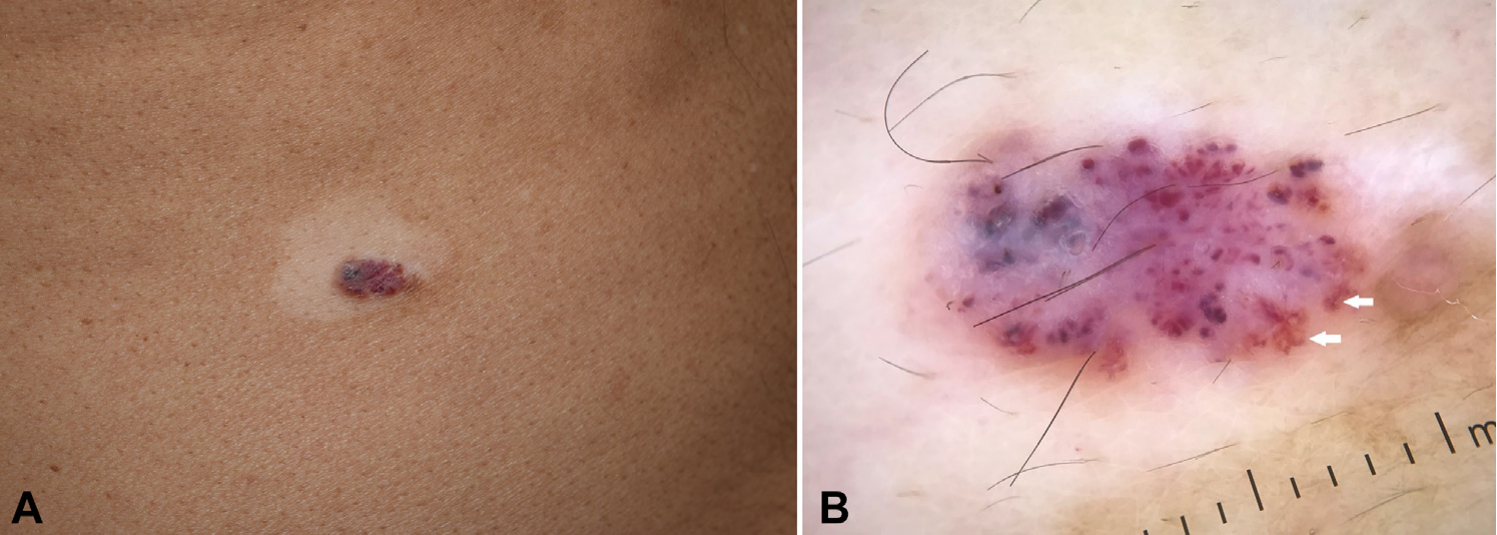
Halo angiokeratoma. **A,** Depigmented halo around a 1 × 0.5-cm dark red plaque on the right flank. **B,** Dermatoscopic examination shows multiple red-to-purple lacunae and whitish veil (DermLite DL4; original magnification: ×10). Small peripheral focal areas of pigmentation can be seen (*arrows*).

A 63-year-old man presented with generalized nonsegmental vitiligo of 10 years’ duration. He is known to have bronchial asthma, diabetes mellitus, hypertension, and dyslipidemia. Examination showed generalized vitiligo (body surface area, 6%) and a depigmented halo around a 1 × 0.5-cm dark red plaque on the right flank (Fig 1).

**Fig 2:**
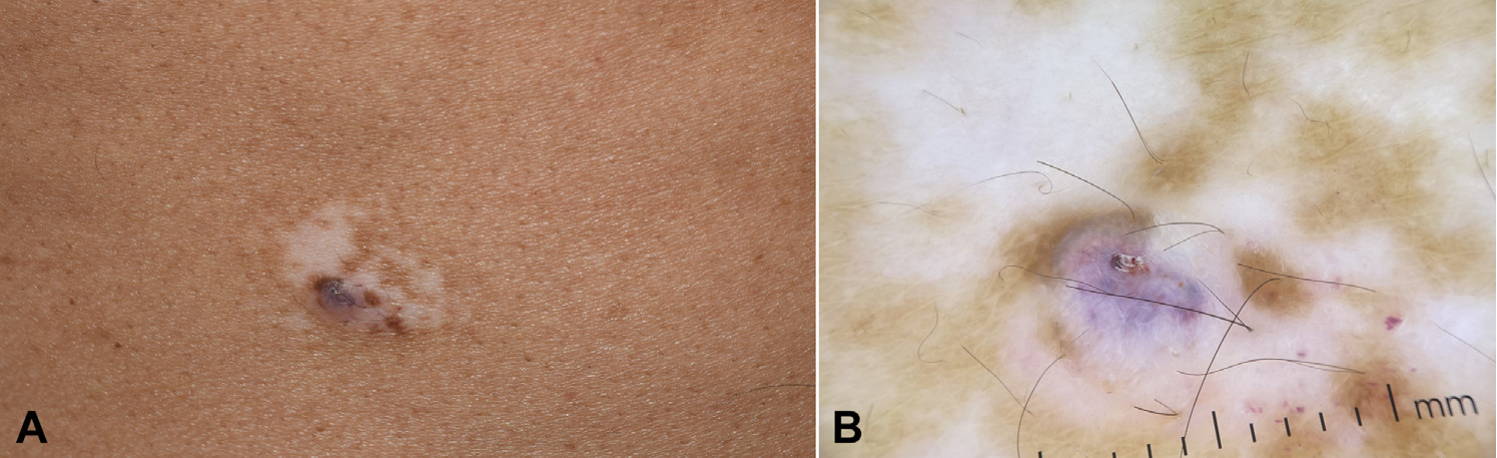
Halo angiokeratoma. **A** and **B,** Clinical and dermatoscopic images taken 2 years after the initial presentation. The angiokeratoma shows a significant reduction in size along with 75% repigmentation of the vitiligo halo. There is a small erosion in the 12-o’clock position of the angiokeratoma dermatoscopic image.

The patient stated that he had this lesion for 15 years. It has never changed or been treated. The white halo developed around the lesion after all other vitiligo patches appeared. Dermatoscopic examination revealed multiple red-to-purple clods and whitish veil (Fig 1). Based on the above clinical and dermatoscopic findings, a clinical diagnosis of halo angiokeratoma was made. The patient was reassured, and only observation of the lesion was recommended. Treatment with tacrolimus 0.1% ointment and narrowband UV-B phototherapy was started for vitiligo. Two years later, a good overall response to therapy was noted for vitiligo. The angiokeratoma showed a significant reduction in size along with 75% repigmentation of the depigmented halo (Fig 2).

**Fig 3:**
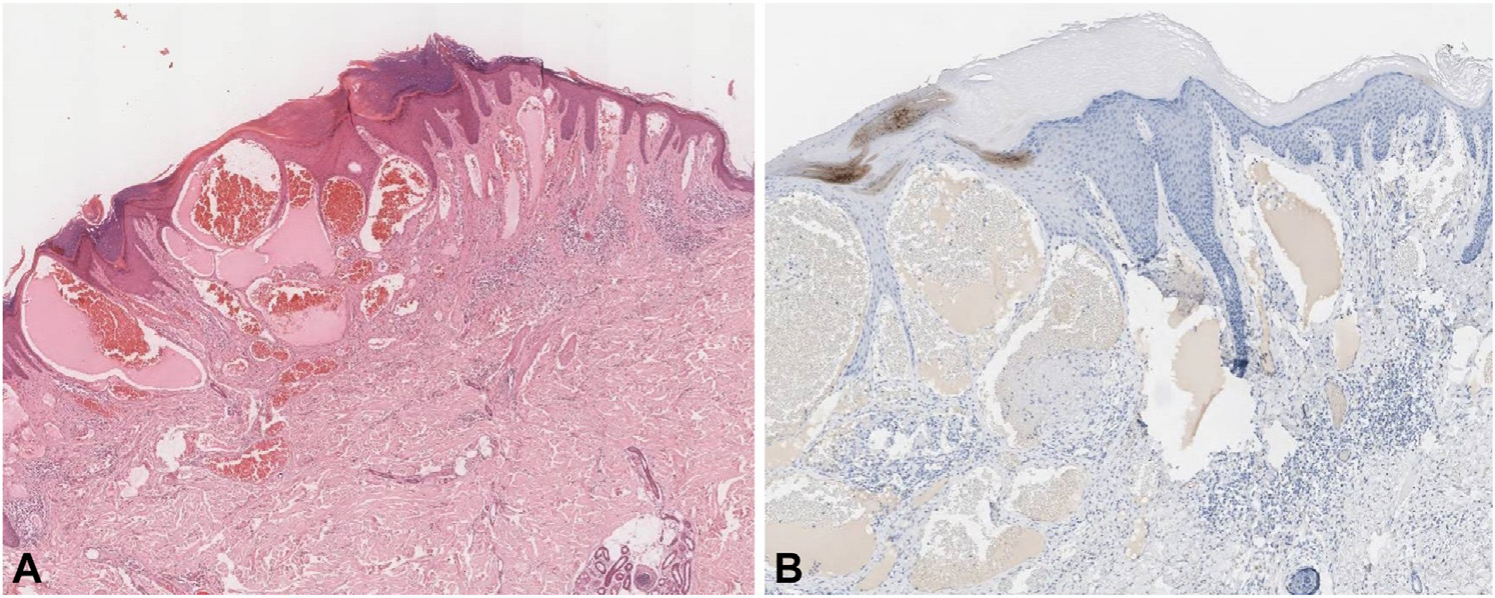
Halo angiokeratoma. **A,** Epidermal hyperplasia with dilated dermal capillary channels and moderate dermal lymphocytic infiltrate (hematoxylin-eosin stain; original magnification: ×20). **B,** Complete absence of melanocytes in the epidermis (Melan-A stain; original magnification: ×40).

The lesion was fully excised and showed histologic features consistent with angiokeratoma associated with absence of melanocytes and moderate dermal lymphocytic infiltrate (Fig 3).

## Discussion

HN occurs because of an immune attack of nevus cells and surrounding epidermal melanocytes by CD8^+^ T cells.^2^ This leads to peripheral depigmentation that is very similar to what is seen in vitiligo. HN can develop alone or in association with vitiligo. In the study by van Geel et al,^3^ isolated HN was associated with lower rates of autoimmune diseases, family history of vitiligo, and Koebner phenomenon compared with vitiligo. However, the number of HN in isolated cases was more than that in those with vitiligo-associated HN. In patients with vitiligo, HN developed before vitiligo onset in the majority of patients. Compared with those without HN, in vitiligo patients with HN, vitiligo developed earlier in life and the patients were less likely to have comorbid autoimmune diseases. In another study, earlier onset of vitiligo and trunk involvement were found to be independently associated with the presence of HN.^4^

The depigmented halo phenomenon can be seen in a wide range of melanocytic nevi. It is mostly observed around junctional, compound, or intradermal nevi. The majority show no or minimal atypia.^5^ Nonmelanocytic lesions were reported less frequently to have the halo phenomenon. There seems to be no reports of angiokeratoma cases to our knowledge.

Two subtypes of white halos around vascular lesions were noted from our analysis of the cases described in the literature: pale halos and depigmented halos. Pale halos around vascular lesions seem to be more common. In a study of 488 patients with cherry angiomas, a pale halo was found in 5.1%.^6^ This perilesional pale halo can also be seen in various vascular lesions because of changes in blood flow, which is attributed to the steal phenomenon.^7^

Depigmented halo around vascular lesions is rarely reported. Kocabaş et al^8^ reported a case of cherry angioma with depigmented halo in an 82-year-old woman. Dermatoscopy showed classic features of cherry angioma. Neither the patient nor her family had vitiligo. Histology evaluation of the lesion was not conducted.^8^ Amico et al^9^ reported an interesting case of telangiectatic melanocytic nevi with depigmented halo in a patient with vitiligo. They clinically appeared as classic cherry angiomas, but histologic evaluation showed dermal melanocytes surrounded by lymphocytes with reticular dermal dilated vessels.^9^ There could have been dermal melanocytes in our case that disappeared because of the immune response and were not available at the time of excision. There were dermatoscopic small peripheral focal areas of pigmentation (Fig 1) that might suggest the co-occurrence of melanocytic proliferation. Finally, Meyerson phenomenon was reported in a case of angiokeratoma.^10^ Although Meyerson phenomenon is characterized by a ring of erythema, it shares with the depigmented halo phenomenon the fact that both are caused by circumferential perilesional inflammation.

A rare finding in our case is the spontaneous reduction in size of angiokeratoma. This can be explained by the associated immune response that leads to disappearance of classic HN with time.^3^^,^^4^ It can also be due to a minor trauma to the lesion that induced an inflammatory reaction as evident by the presence of a small erosion in Fig 2. Interestingly, the regressive changes occurred after initiating therapy for vitiligo. The immunomodulatory effect and regimentation induced by topical tacrolimus and phototherapy could have played a role.

In conclusion, we report a rare case of angiokeratoma associated with a depigmented halo (halo angiokeratoma) with spontaneous shrinkage over time. Although the cause of this phenomenon is not clear, focal presence of melanocytic proliferation might play a role. Finally, this interesting association can simply be due to coincidental colocalization of vitiligo and angiokeratoma. However, this is less likely because there were no other vitiligo lesions affecting the right flank.

## Conflicts of interest

None disclosed.
